# Learning not to feel: reshaping the resolution of tactile perception

**DOI:** 10.3389/fnsys.2013.00029

**Published:** 2013-07-04

**Authors:** Mohsen Omrani, Armin Lak, Mathew E. Diamond

**Affiliations:** ^1^Tactile Perception and Learning Lab, International School for Advanced Studies-SISSATrieste, Italy; ^2^School of Cognitive Sciences, Institute for Research in Fundamental Sciences (IPM)Tehran, Iran; ^3^Centre for Neuroscience Studies, Queen's UniversityKingston, ON, Canada; ^4^Department of Physiology, Development and Neuroscience, University of CambridgeCambridge, UK

**Keywords:** fingertip, human psychophysics, tactile, vibration, learning

## Abstract

We asked whether biased feedback during training could cause human subjects to lose perceptual acuity in a vibrotactile frequency discrimination task. Prior to training, we determined each subject's vibration frequency discrimination capacity on one fingertip, the Just Noticeable Difference (JND). Subjects then received 850 trials in which they performed a same/different judgment on two vibrations presented to that fingertip. They gained points whenever their judgment matched the computer-generated feedback on that trial. Feedback, however, was biased: the probability per trial of “same” feedback was drawn from a normal distribution with standard deviation twice as wide as the subject's JND. After training, the JND was significantly widened: stimulus pairs previously perceived as different were now perceived as the same. The widening of the JND extended to the untrained hand, indicating that the decrease in resolution originated in non-topographic brain regions. In sum, the acuity of subjects' sensory-perceptual systems shifted in order to match the feedback received during training.

## Introduction

When two inputs evoke sufficiently different neuronal responses, our sensory-perceptual systems recognize two distinct events; in contrast when two inputs evoke similar neuronal responses, we perceive two instances of the same event. Behavioral conditions might favor one or the other operation. How do our sensory-perceptual systems learn to parse our experience with the optimal degree of resolution? Perceptual acuity is not purely innate but, rather, is shaped by experience. Thus, by extensive training an expert can make distinctions to which a novice is blind. Under laboratory conditions, a sensory system can be “tuned” to recalibrate and to perform progressively finer discriminations of visual stimuli (Herzog and Fahle, 1997; Dill and Fahle, 1998), auditory stimuli (Kishon-Rabin et al., 2001; Bosnyak et al., 2004) or tactile stimuli (Recanzone et al., 1992a,b; Sathian and Zangaladze, 1997; Ostwald et al., 2012), temporal events or even multisensory stimuli (Fujisaki et al., 2004; Vroomen et al., 2004; Keetels and Vroomen, 2008; Yamamoto et al., 2012). In many of the cases cited above, recognition of small differences between stimuli was rewarded and, consequently, subjects showed improvements in sensory resolution.

Here we set out to test the complementary (and less intuitive) hypothesis—that sensory systems can be trained to lose discriminative resolution. Unlike previous studies, one set of subjects was rewarded during training for classifying distinguishable somatosensory stimuli as being the same. During training, their rewards could thus be maximized by a loss of resolution in the sensory-perceptual system; by broadening rather than sharpening the categories of discriminable events. Before and after training, we tested the subjects' sensory discriminative capacities to investigate whether biased feedback had influenced the subjects' perceptual acuity. The findings suggest that both improving and lessening discriminative capacity might involve a single underlying mechanism, one that can achieve higher or lower perceptual acuity according to the feedback given to the sensory system.

Having found changes in the resolution of sensory discrimination, we asked at what level of the sensory-perceptual system those changes occurred. If the modifications were in a strictly topographic representation such as the primary somatosensory cortex, we would expect subjects to show the altered categorization only when tested with the fingertip that received training. If the modifications involved a change in the “read out” of the activity of the topographic representation, we would expect the altered categorization to extend to a broader set of sensory inputs. To gain insight into this problem, we tested one set of subjects with the fingertip opposite the trained one.

## Results

### Overview

**Figure 1:**
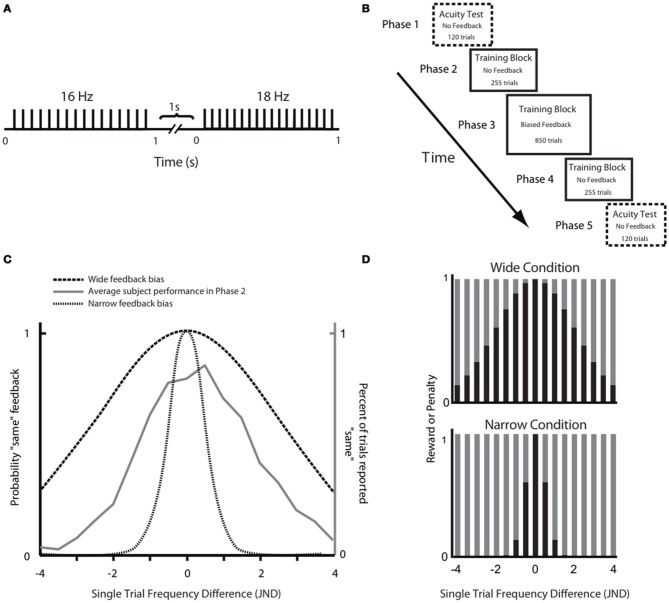
**Experimental paradigm. (A)** Schematic representation of a stimulus pair. Each vertical bar indicates a 2.5 ms half-sinusoidal deflection by the piezoelectric stimulator. Stimulus frequency was determined by the peak-to-peak inter-deflection interval. In acuity tests [Phases 1 and 5; see panel **(B)**] the first and second stimuli were both 1 s long. In the training session (Phases 2, 3, and 4), the first and second stimuli were 1 and 2 s long, respectively. **(B)** Sequence of phases. For each phase, the presence or absence of feedback is given as well as the number of trials. **(C)** Comparison of Wide and Narrow feedback distributions to average subject response curve. The gray line shows, for Phase 2, the proportion of trials in which subjects, on average, reported two stimuli to be “same” as a function of the single trial frequency difference. The two dashed black lines show, for Phase 3, the proportion of trials in which the computer gave “same” feedback as a function of the single trial frequency difference in the Wide and Narrow conditions. **(D)** Reward size in the Wide condition (top) and the Narrow condition (bottom) varied according to the probability of the computer giving “same” and “different” feedback in each condition. Each bar illustrates the probability a priori of the computer providing that answer (black, same; gray, different). In each condition, the reward for the correct “same” responses was proportional to the sum of all gray bars while the reward for the correct “different” responses was proportional to the sum of all black bars.

Our overall aim was to test the hypothesis that a paradigm that rewarded subjects for judging discriminable stimuli as being the same would diminish the subjects' sensory acuity. In other words, we wanted to find out whether the brain can learn “not to feel” previously detectable stimulus differences. Throughout the experiment subjects were asked to compare the frequencies of trains of skin deflections, presented as pairs (Figure 1A). Through an extensive training phase (Figure 1B), subjects received feedback on each trial in the form of points given to them or taken away depending on whether their choice agreed or disagreed, respectively, with the computer's feedback. For subjects in the Wide group, the computer's feedback was biased in order to reward them for judging discriminable stimuli as being the same; subjects in the Narrow group received feedback that rewarded their ability to discriminate stimulus differences (Figure 1C). The perceptual acuity of each subject was tested before and after the training session.

### Just noticeable difference prior to training

**Figure 2:**
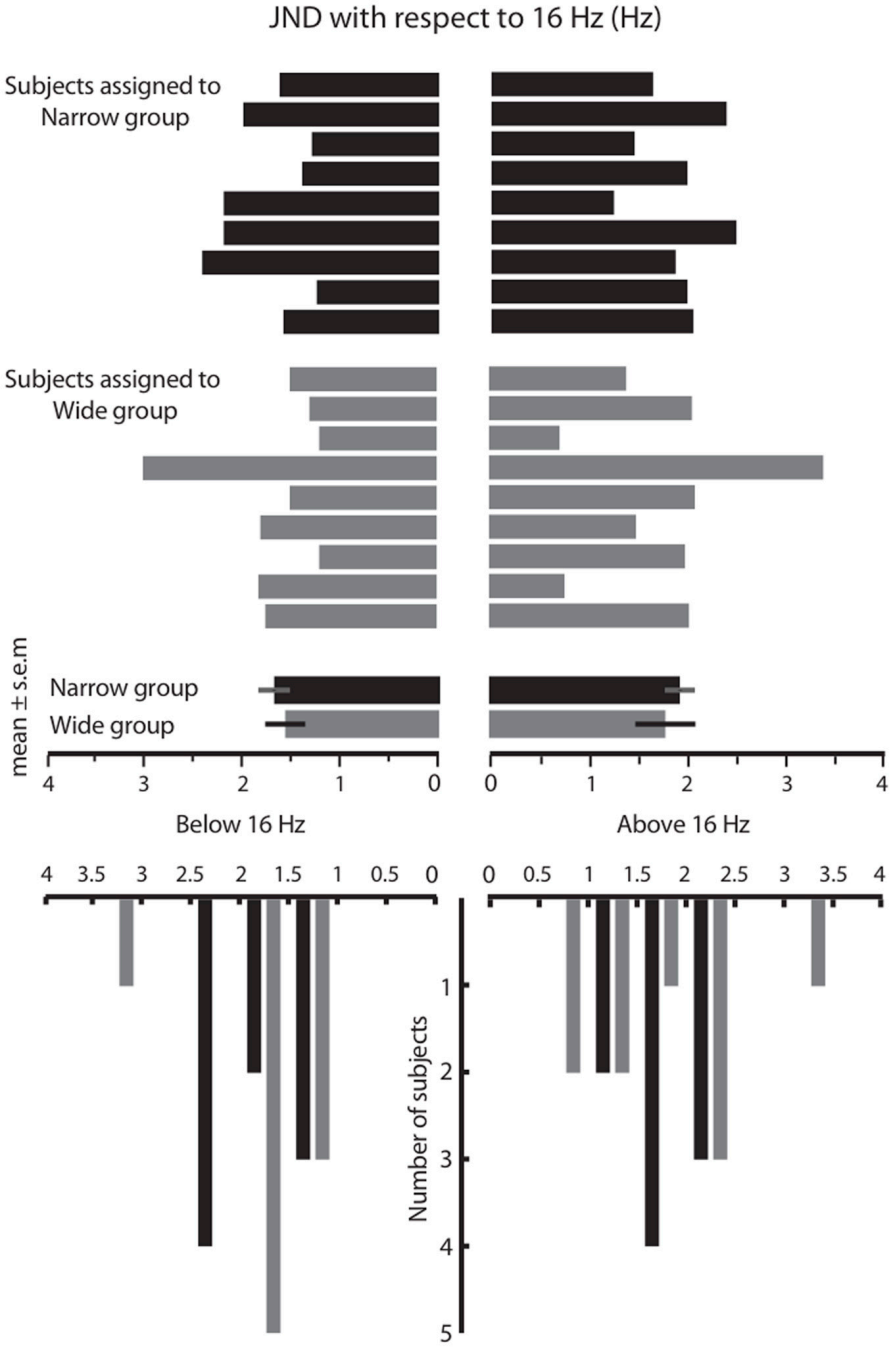
**Results of the acuity test in Phase 1**. The **top** panel shows the just noticeable difference (JND) values for all subjects that participated in the Wide and Narrow conditions, measured separately below 16 Hz (to the left) and above 16 Hz (to the right). The **bottom** panel is formed by counting the number of subjects with JND values in each bin (bin size = 0.5 Hz). Overall, the JND distribution was similar in the two groups prior to the training session.

In Phase 1 of the experiment, we measured the frequency resolution of each subject by using a staircase procedure (see Materials and Methods) to estimate the just noticeable difference (JND) above and below 16 Hz. The results are given in Figure 2. The above-16 JND values ranged from 0.7 to 3.4 Hz, with a median of 2.0 Hz. The below-16 JND values ranged from 1.2 to 3 Hz, with a median of 1.6 Hz. The distribution of JNDs above 16 Hz and below 16 Hz did not differ significantly (Wilcoxon signed rank, *p* = 0.33). Henceforth, we pooled the above and below 16 Hz JNDs for each subject and used the absolute JNDs for further analysis. The overall distributions of JND values were similar in the two groups prior to training (Figure 2). The median JND value was 1.65 for the Wide group and 1.72 for the Narrow group (Wilcoxon signed-rank test, *p* = 0.29). The JND in our subjects, approximately 10% of the base stimulus, was comparable to that reported in previous studies of vibrotactile discrimination in humans and monkeys (Goff, 1967; LaMotte and Mountcastle, 1975; Mountcastle et al., 1990; Recanzone et al., 1992a,b).

Phases 2 and 4 each consisted of 255 trials, across which subjects had to compare a 16 Hz stimulus to 17 different stimuli. These stimuli deviated from the reference 16 Hz stimuli by half-JND increments (eight frequencies above and eight below 16 Hz, as well as 16 Hz itself). We refer to the distance between 16 Hz and the second frequency presented on any given trial as the “single trial frequency difference” (STFD). Subjects reported whether the two frequencies felt the “same” or “different” on every trial; there was no feedback and no points awarded. The second column of Table 1 gives the percent of trials that were judged as “different” in Phase 2 for selected single trial frequency differences (STFDs), averaged across subjects. Comparing these values to the probabilities of “same” and “different” feedback in Phase 3, it is evident that the computer's feedback for two perceptually separable stimuli would mostly be “same” in the Wide condition (Table 1, columns three and four) and “different” in the Narrow condition (Table 1, columns five and six). Of course, our inference of the mismatch between percept and feedback in the Wide condition is based on the subjects' acuity at the start of Phase 3. The results given below will show that, by the end of Phase 3, the mismatch had been largely resolved by a change in sensory acuity.

**Table 1:** **Feedback parameters for subjects in the wide and narrow condition**.

**Single trial frequency difference (STFD) in multiples of just noticeable difference (JND)**	**Phase 2: Percent of trials in which stimuli were judged as “different” (average of all subjects) (%)**	**Phase 3: Wide condition**		**Phase 3: Narrow condition**	
		**Probability “same” feedback**	**Probability “different” feedback**	**Probability “same” feedback**	**Probability “different” feedback**
0	15	1.0	0	1.0	0
0.5	18	0.93	0.07	0.60	0.40
1.0	32	0.87	0.13	0.13	0.87
2.0	67	0.60	0.40	0.06	0.94
4.0	94	0.13	0.87	0	1.0

### Response distributions before and after biased feedback

**Figure 3:**
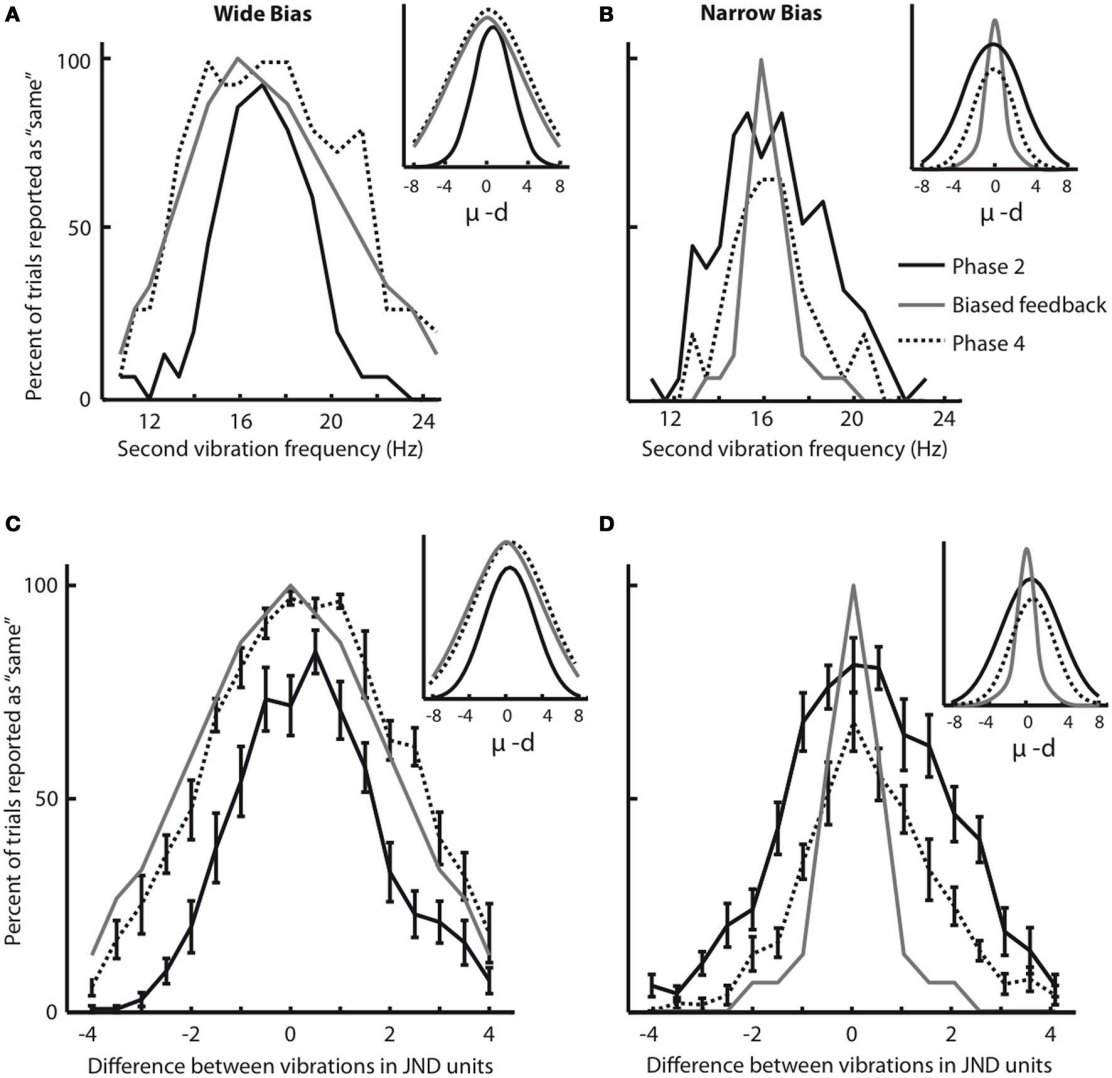
**Changes in response distribution caused by feedback. (A)** Response distribution (proportion of “same” responses across frequency pairs) for a subject that received Wide feedback. Distribution in Phase 2 (before feedback) is shown in black. The feedback distribution in Phase 3 is shown in light gray. Similar to Figure 1C, the feedback curve illustrates the fraction of trials in which the “same” feedback was provided. The response distribution in Phase 4 (after feedback) is shown by dotted line. Inset illustrates Gaussian functions fitted on the response distributions. **(B)** Response distribution for a subject that received Narrow feedback. The conventions for the lines are the same as in **(A)**. Inset illustrates Gaussian functions fitted on the response distributions. Although the subjects in **(A)** and **(B)** showed similar distributions in Phase 2, by Phase 4 they differed noticeably. **(C)** Response distributions averaged across subjects that received Wide feedback. The conventions for the lines are the same as in **(A)**. The error bars indicate standard error of mean (SEM) across subjects. Inset illustrates Gaussian functions fitted on the response distributions. **(D)** Response distributions averaged across subjects that received Narrow feedback. The conventions for the lines are the same as in **(A)**. Note that each subject received equal number of same/different feedback in each condition as the feedback was drawn from a pseudorandom pool. Insets illustrate Gaussian functions fitted on the response distributions. Although the subjects in **(C)** and **(D)** showed similar distributions in Phase 2 (Wilcoxon rank-sum test, *p* = 0.1), by Phase 4 they differed noticeably.

Phases 2 and 4 of the experiment allowed us to construct response distributions in which we evaluated the performance of each subject, across all frequency pairs, in the absence of any feedback. The response distribution is composed of the percent of trials for which the subject judged a stimulus to be the “same” as the 16 Hz stimulus, as a function of its difference from 16 Hz. Figure 3A shows the results for a subject that participated in the Wide condition. The solid black plot gives the response distribution in Phase 2. The gray plot shows the Wide distribution from which feedback was drawn during Phase 3 of training (the asymmetry in the feedback curve is due to a small difference in the subject's under-16 and over-16 JNDs). The dashed plot shows the subject's response distribution in Phase 4, the feedback-free block that followed the biased feedback period. It is evident that the subject's response distribution became similar to the feedback curve.

Figure 3B uses the same convention to present the results for a subject that participated in the Narrow condition. In Phase 4, the subject's response distribution approached the Narrow feedback curve. The sets of sensory stimuli presented to the two subjects were identical; the difference was limited to the feedback provided in Phase 3.

To quantify the magnitude of response distribution adaptation, a Gaussian function (Equation 1 in Materials and Methods) was fit to each response distribution. Figures 3A,B (insets) demonstrate the fitted Gaussian curves for the selected subjects (Figure 3A: Gaussian goodness of fit *r* = 0.98 and 0.95 in Phases 2 and 4, respectively; Figure 3B: Gaussian goodness of fit *r* = 0.95 and 0.96 in Phases 2 and 4, respectively). The breadth of the response distribution, σ, increased in the Wide condition (Figure 3A: σ = 2.12 in Phase 2 and σ = 4.62 in Phase 4. Note that σ is defined in half-JND steps. Hence, in this example σ increased from 1.06 * JND to 2.31 * JND). On the other hand, the breadth of the response distribution decreased in the Narrow condition (Figure 3B: σ = 3.22 in Phase 2 and σ = 2.12 in Phase 4) in accordance with the biased feedback provided during Phase 3. In the Wide condition, σ in the Phase 4 response distribution matched σ of the feedback distribution. In the Narrow condition, the response distribution in Phase 4 approached the σ of the feedback distribution but remained slightly wider.

We carried out the same analysis for the entire set of subjects. To be able to combine the data from subjects with different baseline sensitivities (i.e. JND), all stimuli were translated to their distance from 16 Hz, normalized as JND steps. Figure 3C shows the Phase 2 (solid black lines) and Phase 4 (dotted black lines) response distributions averaged across all subjects in the Wide group; Figure 3D shows the comparable data for the Narrow group. These panels also show the average feedback distributions (light gray lines). Several observations can be made. The average Phase 2 response distributions were nearly the same for the subjects in the Wide and Narrow groups; this follows from the fact that, in Phase 2, the two sets of subjects had not yet received their differential feedback biases. It is also clear that, for the Wide group, the response distribution in Phase 4 overlaid the Phase 3 feedback distribution. In other words, subjects on average reported stimulus pairs as being “same” with the same probability that the computer-generated feedback reported “same.” For the Narrow group, the response distribution in Phase 4 approached the Phase 3 feedback distribution. Overall, the Narrow feedback had the effect of reducing the subjects' probability of giving the “same” responses.

Gaussian functions (Figures 3C,D insets) were fit to Phase 2 and Phase 4 response distributions of all subjects; mean goodness of fit across all subjects was *r* = 0.93 for both Phases 2 and 4 in both Wide and Narrow groups. We compared values of the Gaussian parameters for the two conditions. In Phase 2 (before receiving biased feedback) as expected the breadth of the response distribution, σ, did not differ for subjects assigned to the either of the groups (Wilcoxon rank-sum test, *p* = 0.1). In the Wide condition, the standard deviation of the fitted functions, σ, increased significantly following the biased feedback of Phase 3 (mean σ in Phase 2 = 2.82, mean σ in Phase 4 = 4.09; Wilcoxon signed rank test, *p* < 0.005). In Phase 2, σ of the response distribution was significantly smaller than that of the feedback distribution that was to be applied in Phase 3 (Wilcoxon rank-sum test, *p* < 0.001). However, in Phase 4 there was no significant difference between σ of the response distribution and that of the just-concluded feedback distribution (*p* = 0.35). This means that subjects adapted their response distributions to match the feedback distribution. In the Narrow condition the breadth of the fitted functions, σ, decreased significantly following the biased Narrow feedback of Phase 3 (mean σ in Phase 2 = 3.28, mean σ in Phase 4 = 2.35; Wilcoxon signed rank test, *p* < 0.005). In Phase 2, σ of the response distribution was significantly broader than that of the upcoming feedback distribution (Wilcoxon rank-sum test, *p* < 0.001), but unlike the Wide condition, the value of σ in Phase 4, did not match σ of the preceding feedback distribution (*p* < 0.005). Thus, the average performance of the subjects in the Narrow condition resembled that of the selected example (Figure 3B): the average response distribution in Phase 4 was narrower than that in Phase 2, but not as narrow as the “target” feedback distribution of Phase 3.

### Changes in perceptual acuity after biased feedback

**Figure 4:**
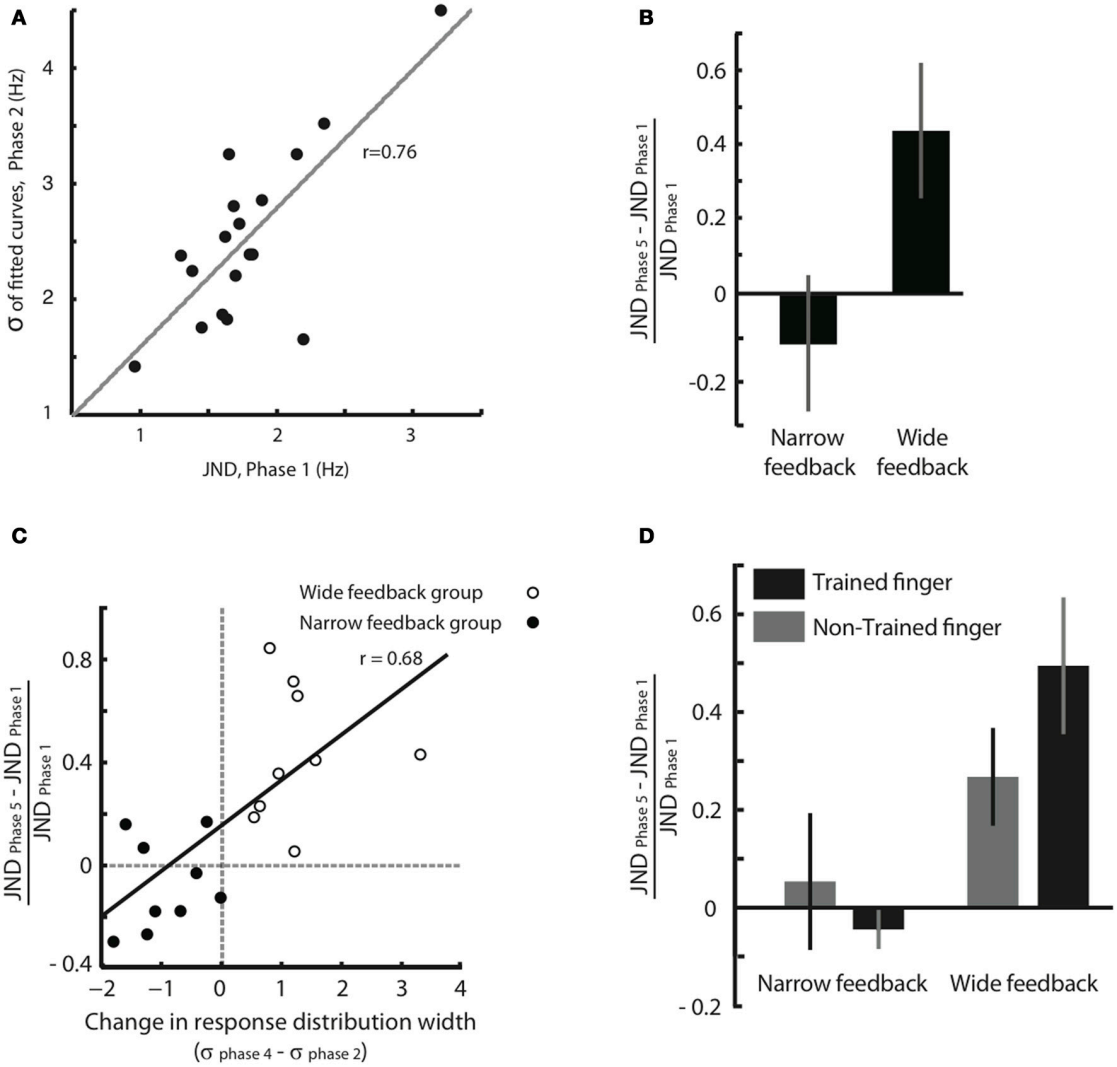
**Effect of feedback on tactile acuity. (A)** Width of the response distribution in Phase 2 (quantified by the standard deviation, σ, of the fitted Gaussian curve) plotted against the JND obtained moments earlier, in Phase 1. **(B)** Normalized JND change (difference in each subject's JND between Phases 5 and 1, divided by the JND of Phase 1) following training paradigm with Wide and Narrow biased feedbacks. Error bars are SEM across subjects. **(C)** The correlation between normalized JND change (Phase 5 compared to Phase 1) and the difference in response distribution breadth (standard deviation, σ, of the fitted Gaussian curve in Phases 4 compared to that in Phase 2). There is a significant correlation between changes in breadth and changes in JND that extends across both the Narrow and Wide feedback groups. **(D)** Changes in perceptual acuity, quantified as the normalized JND change, for both the trained finger and the opposite, untrained finger. Subjects with Narrow feedback are given on the left, and no significant change in JND of the untrained finger was observed. Results for subjects with Wide feedback are given on the right, where a significant increase in JND of the non-trained finger was observed. Values for the trained finger, for comparison, are reproduced from panel **(B)**. The error bars indicate SEM across subjects.

We think that the response distribution (Figure 3) and the JND provide two alternative methods for tapping into a single perceptual process—the acuity in feeling frequency differences. To test this notion, in Figure 4A we plotted for all individual subjects the broadness of the response distribution in Phase 2 against the JND obtained moments earlier, in Phase 1. The response distribution breadth was calculated in the original units of Hz rather than in units of JND. The high correlation between the two measures (correlation coefficient *r* = 0.76, *p* < 0.001) supports the idea that JND and response distribution breadth together reflect the same underlying function.

The results given in Figure 3 demonstrate that the subjects' judgment of stimuli in the neighborhood of 16 Hz was shaped by feedback during training. However, since the training paradigm involved a “same/different” comparison, a change in the decision criterion—rather than a change in perceptual acuity—might be the cause of the shift in the response distribution. To obtain a second, more direct measure of change in acuity, we calculated and compared JND before and after training (Phase 1 vs. Phase 5). Figure 4B shows that, in the Wide condition, the JND significantly increased (median normalized JND change among all subjects = 0.40, Wilcoxon signed rank test, *p* = 0.001). In the Narrow condition, the JND showed a trend suggesting a decrease, but the change was not statistically significant (median normalized JND change among all subjects = −0.13, Wilcoxon signed rank test, *p* = 0.23). These results indicate that, departing from a baseline state, subjects can be more robustly trained to lose acuity than to gain acuity.

During Phase 3, subjects might notice that one form of feedback was predominant—“different” in the Narrow group and “same” in the Wide group. At that point, subjects might reduce their attention to the specific stimuli on each trial, and instead provide the “easy” answer (i.e., the more prevalent form of feedback). It cannot be ruled out a priori that such a process could have led to the broadening of the response distribution in Wide subjects, and the narrowing of the response distribution in Narrow subjects. However, there are three arguments that the changes in response distribution reflected a true change in perceptual acuity. First, to prevent decisions based on feedback prevalence, in Phase 3 the number of points awarded for each response was inversely related to the probability of the computer providing that answer (Figure 1D). Therefore, the expected award for each answer (the product of the probability of that answer matching the feedback and the award amount for that answer if correct) was equal for “same” or “different” answers in each condition. For instance, if a subject decided to use the easy, matching strategy (e.g., always report “different” in the Narrow condition), the number of correct answers might rise, but the points collected would be small. Indeed, as we punished the subjects for wrong choices, the collected award might become negative (+3 point for correct “different” choice and −9 points for wrong “different” choice). Second, in Phases 2 and 4 there was no feedback. The subjects' altered response distributions (Figure 3) presumably reflected their percept, in as much as there was no feedback to aim for.

The third and most important argument comes from within-subject comparison of response distribution change and JND change (Figure 4C). The plot confirms that subjects in the Wide group (unfilled circles) showed an increase in the width of the response distribution, as well as an increase in JND; subjects in the Narrow group (filled circles) tended to show a decrease in the width of the response distribution, as well as a decrease in JND. The critical point is that there was a strong within-subject covariance between response distribution change and JND change (Pearson correlation coefficient *r* = 0.68, *p* = 0.001). This correlation is incompatible with the notion that subjects simply changed threshold across Phases 2, 3, and 4 in order to match the computer's biased feedback. Rather, the data suggest that response distribution breadth and JND tap into the same underlying acuity mechanism. Any sharpening or broadening of this discrimination capacity was expressed in both the “same/different” task of Phases 2–4 and the “high/low, low/high” task of Phases 1 and 5.

### Changes in the perceptual sensitivity of untrained finger

Having found changes in the resolution of sensory discrimination, we asked at what level of the sensory-perceptual system those changes occurred. If the modifications were in a strictly topographic representation such as the primary somatosensory cortex, we would expect subjects to show the altered categorization only when tested with the fingertip that received training. If the modifications involved a change in the way activity in the topographic representation was “read out” to make perceptual judgments, we would expect the altered categorization to extend to a broader set of sensory inputs. To gain insight into this problem, we carried out a second experiment in which subjects were tested with the fingertip opposite the trained one. For the trained fingertip, Phases 1–5 were all presented. For the untrained fingertip, only Phases 1 and 5 were carried out.

For subjects that received training in the Narrow condition, no change in JND of the non-trained hand was observed (Figure 4D, normalized JND change = 0.05, Wilcoxon signed-rank test, *p* = 0.74). This result was expected since, as in Experiment 1, the Narrow condition caused only a moderate change in JND even for the trained finger (normalized JND change = −0.04, Wilcoxon signed-rank test, *p* = 0.31). In contrast, for subjects that received training in the Wide condition, a significant increase in JND of the non-trained hand was observed (normalized JND change = 0.27, Wilcoxon signed-rank test, *p* = 0.039), just as it was for the trained finger (normalized JND change = 0.49, Wilcoxon signed-rank test, *p* = 0.015). Since the decrease in acuity took place for the finger that received no training, the changes in sensitivity must not be localized to primary somatosensory cortex but must arise in a “downstream” region that receives input from both hands. We suggest that such a downstream area, like SII, decreases the resolution with which it decodes SI activity during training with Wide feedback.

## Discussion

This study looked for changes in individual subjects' acuity in relation to the form of feedback employed during training. In each experiment, either Wide or Narrow feedback was applied. The Wide form of feedback entailed a high probability of the computer reporting the stimulus pair as the “same” even in trials in which the difference between stimuli was larger than the individual subject's JND and therefore distinguishable; the Narrow form of feedback entailed a high probability of the computer reporting the stimulus pair as “different.” The first form of feedback thus tended to reward the grouping of different stimuli into the same perceptual category, while the second rewarded subjects for exploiting their finest possible perceptual acuity. The main result is the change in the frequency discrimination capacity between Phase 1 and 5. Although the Wide and Narrow subject groups received the exact same set of sensory stimuli (normalized to their JND)—the only difference in training being the feedback following each trial during Phase 3—the JND grew in subjects that experienced the Wide training condition. One might predict by symmetry that the JND would contract in subjects that experienced the Narrow training condition. In some subjects the predicted contraction did occur, but taken as a group the effect did not reach statistical significance (*p* = 0.23). We hypothesize that even before training subjects were near the verge of their physiological limits for sensory acuity. If the default state of the sensory system at the experiment outset was close to its high-resolution state, a greater number of trials than used here might be necessary to produce significant acuity improvements across all subjects (Sathian and Zangaladze, 1998; Grant et al., 2000; Harris et al., 2001a,b,c).

While these result suggest a general change in the tactile representation of the stimuli, our findings do not specify whether such changes are restricted to the representation of the core frequency (16 Hz) or else are distributed throughout neighboring frequencies. Testing the distribution would require repeating the acuity test for each neighboring frequency separately. Another question for further work is whether changes in acuity can be evoked in an asymmetrical manner (e.g., widening of acuity above 16 Hz and sharpening of acuity below 16 Hz).

### Progressive loss of tactile acuity during biased feedback

During Phase 3, the first period in which subjects received biased feedback, shifts in sensitivity emerged. We quantified this initial shift in acuity as a change in the width of the response distribution (Figure 3). The distribution became wider in subjects undergoing the Wide training condition, and tended to become narrower in subjects undergoing the Narrow training condition, though the effect was less substantial in the latter case. An adaptation of this sort is not by itself a convincing demonstration of a change in sensory processing: subjects in the Wide condition might realize that feedback favors the “same” response and might attempt to match the feedback by giving the “same” response even on trials where they receive two discriminable vibrations. By the same token, subjects in the Narrow condition might realize that the feedback favors the “different” response and might tend to provide the corresponding response on all trials, even when the stimulus difference was below JND. Such adjustments would constitute shifts in threshold, not shifts in acuity. In fact, a recent paper (Engel and Wang, 2011) presents a recurrent neural network which shows similar changes in same/different comparison distribution given the probability of a favorable answer (for instance “different” in the Narrow condition) or the reward assigned to each answer.

The fundamental issue, then, is to specify whether subjects' answers began to mirror the bias in feedback due to a change in the categorization threshold, or through a real change in perceptual acuity. To distinguish between these possibilities, in Phases 1 and 5 we measured JND using a forced choice paradigm, where subjects had to report two successive vibrations as having either a high/low or low/high sequence. This paradigm excludes the strategy of comparing the perceived difference between the stimuli to some internal criterion for sameness or differentness. Moreover, during the assessment of JND subjects received no feedback so there could be no gain by attempting to match a feedback bias. Having ruled out the possibility that the increase in JND in the Wide condition was accounted for by a criterion change, the remaining explanation is a progressive loss of acuity: stimulus differences that previously exceeded the JND could no longer be detected. In summary, at some level of the brain the representations of two stimuli overlapped more than they did prior to training.

### Experience-dependent setting of perceptual boundaries

The shifting or even disappearance of a perceptual boundary is known in sensory modalities beyond touch (Li et al., 2008). In speech, listeners detect a boundary along the continuum between consonants, a phenomenon called categorical perception (Liberman et al., 1957). For instance, between /r/ and /l/, native English speakers perceive sounds on one side of the boundary as /r/ (as in “road” or “bread”) and on the other side as /l/ (as in “load” or “bled”). Tests of discrimination show that the continuum is consistently divided into two phonetic categories; across-category pairs of sounds are distinguished well but within-category pairs are distinguished poorly (MacKain et al., 1981).

However this boundary is lost, or never acquired, in native speakers of some other languages, such as Japanese. These subjects discriminate between sound pairs along the continuum in a nearly random manner and perform no better for comparisons across the English phonetic boundary than for comparisons within either the /r/ or the /1/ category (MacKain et al., 1981). The existence or non-existence of the boundary is stabilized by the age of 10–12 months.

Perceptual discriminations are thus not built into sensory processing systems in a hard-wired manner, but can be formed and shaped by experience (Maddox, 2002). While it can be debated whether a sensitivity change in the realm of tactile perception involves similar mechanisms to those underlying language, it is important to note that plasticity analogous to the present work has been reported in sensory-motor adaptation. For instance, it has been shown that following motor adaptation, the sense of position is re-calibrated to reflect the dynamics of the motor task (Ostry et al., 2010; Mattar et al., 2011; Henriques and Cressman, 2012). This recalibration is accompanied by a change in the magnitude of sensory evoked potentials (Nasir et al., 2013), suggesting a change in somatosensory coding. The present study reveals that a change in perceptual discrimination can be evoked simply through non-veridical feedback. Interestingly, the change in discrimination was achieved within just a few hundred trials, far more rapidly than changes in speech perception.

### Evidence for the brain locus of learning

We found that the broadening of the JND occurred not only for the fingertip to which stimuli had been presented during training, but also for the opposite fingertip. This transfer of the training effect provides clues about which brain regions may be involved. Primary somatosensory cortex (SI) contains a topographically sharp representation of the fingers on the contralateral side of the body with no appreciable input from the ipsilateral hand (Blankenburg et al., 2003; van Westen et al., 2004). The training regime was applied to the right index finger; if changes in left SI were responsible for broadening of the JND, then test stimuli originating on the untrained (left) hand would be transmitted to the right SI and would thus bypass the modified cortical region. This scenario predicts that the training effect would be restricted to the right hand, the one that received the biased feedback, so it cannot account for our results.

In contrast, secondary somatosensory cortex (SII) contains a topographically broad representation of the fingers with comparable strength of input from both the contralateral and ipsilateral sides of the body, predominately by crossed and uncrossed inputs from SI (Gelnar et al., 1998; Diamond et al., 1999; Maldjian et al., 1999; Francis et al., 2000; Disbrow et al., 2001; Harris et al., 2001a,c; Diamond et al., 2003; Harris et al., 2006). If the biased feedback employed in the Wide condition had the effect of reducing the resolution with which SII “reads out” its input from SI, this would explain the transfer of the loss of acuity to the untrained hand. In this scenario, left and right SI stimulus representations are unchanged, but SII develops the incapacity to decode differences in stimulus representation in the vicinity of 16 Hz, whether the sensory signal arrives from left or right SI. This suggests that the observed learning effects are due to a high-level reweighting of perceptual readout rather than modifications in early sensory stage (Sathian et al., 2013). Linear discriminant models offer inspiration for a potential mechanism to decrease the read out resolution. Typically the challenge posed to such a model is to achieve the most informative readout by a target neuron. The solution involves the optimal weighting of all synaptic inputs [reviewed in Safaai et al. (2013)]. An improvement in acuity can be realized by increasing the weights from the more informative neurons and decreasing the weights from the less informative neurons. Wide feedback training causes some level of the sensory system to execute a poorer readout; this reduction in acuity might be accomplished, paradoxically, by increasing the weights from the less informative SI neurons and decreasing the weights from the more informative neurons.

Traditionally, delayed comparisons of vibrotactile frequency have been thought to occur in the sensory areas like SI or SII (e.g., Mountcastle et al., 1990; Harris et al., 2006; or Hernández et al., 2010), with the main discussion focusing on whether more primary sensory areas are involved or more secondary areas. From the topography of the generalization of the widened acuity, to the finger opposite the trained one, we suggest that the change took place in the earliest sensory cortical region that integrates strong bilateral inputs, SII. Of course, future studies may better specify the brain locus of vibrotactile frequency comparison.

### Setting perceptual resolution to optimize matching between choice and outcome

Psychophysical studies have shown that animals and humans can behave as optimal Bayesian observers—they integrate noisy sensory cues, their own predictions and prior beliefs in order to maximize the expected outcome of their actions (Kording and Wolpert, 2004; Ernst, 2007; Navalpakkam et al., 2010; Rao, 2010; Weisswange et al., 2011; Karim et al., 2012). A key to maximizing the outcome is to have knowledge of the certainty of sensory evidence, for higher degrees of uncertainty encourage the nervous system to weaken its belief in the ongoing model and to explore alternative stimulus-outcome associations (Karlsson et al., 2012). It has been suggested that neuronal populations in sensory areas represent external events as “probabilistic population codes” which incorporate both the physical properties of a sensory event as well as the uncertainty associated with that information (Knill and Pouget, 2004; Ma et al., 2006, 2008). Interestingly, the population response in early sensory cortex can be biased in favor of stimuli with higher values (Serences and Saproo, 2010). We posit that early in Phase 3 subjects in the Wide condition received “same” feedback even for stimulus pairs that SI was able to encode as distinct events. The mismatch between the sensory representation and the feedback caused an increase in the degree of uncertainty and facilitated the exploration of alternative modes of “reading out” the sensory input. The mismatch could be resolved to some degree by diminishing the acuity of the transfer of information from SI to SII or from SII to even higher-order cortical areas (perhaps by overweighting the uninformative inputs; see above). In higher-order areas, the neuronal activity correlates with the perceptual report rather than stimulus itself (Auksztulewicz et al., 2012; Carnevale et al., 2012; Romo et al., 2012). The outcome of the reduced acuity was the broadening of the response curves (Figures 3A,C). This in turn led to a better match between the subjects' responses and the computer's feedback. Having matched the feedback, the uncertainty in the sensory readout was again reduced and the modifications became stabilized. The consequence of the stabilization was the bilateral reduction in acuity revealed by tests of the JND at the conclusion of the experiment. Taken together, these findings begin to provide a framework for the brain's capacity to learn not to feel.

## Materials and methods

### Subjects

Fifteen subjects (11 F, 4 M; age range from 21 to 35 years) took part in the experiment. All were right-handed. The overall study consisted of Experiments 1 and 2. All subjects participated in Experiment 1, which was composed of two training conditions, Narrow and Wide. Subjects were randomly assigned to one of the two conditions; Nine subjects took part in the Narrow condition and nine in the Wide condition (three subjects took part in both conditions with a minimum two-week gap between sessions). Out of the pool of 15 Experiment 1 subjects, eight also took part in Experiment 2. Recruitment of subjects and experimental procedures were conducted in accordance with the Declaration of Helsinki and the local Ethics committee of SISSA.

#### Sensory stimuli

The stimulus train was delivered to one fingertip, which rested on a 2 cm × 2 cm rubber pad glued to the top face of a piezoelectric wafer (Morgan Matroc, Bedford, OH). The timing and waveform of the vibrations were controlled using MATLAB (MathWorks, Natick, MA) and a National Instruments (Austin, TX) interface board. Stimulus trains (Figure 1A) were composed of deflections of half-sinusoidal shape, with each deflection having duration of 2.5 ms and peak amplitude of 150 μm. Before experiments began, stimulus output was measured by an optic sensor (Lak et al., 2008, 2010) to verify that it conformed to the waveform input. Stimulus frequency in Hz was equal to 1000 divided by the peak-to-peak interdeflection interval in ms. For different frequencies, the individual pulse remained equivalent and thus engaged the same set of skin receptors. The duration of the stimulus train, from the first to the last deflection, was one period shorter than 1 s (half a period lag at the beginning and half at the end). For instance, for a 16 Hz stimulus, the vibration was presented for 937.5 ms with each deflection (16 in total) separated by 62.5 ms. This was done to allow fractional frequency values, rather than limiting the frequency content to integers. After each trial the subjects reported their response using a keyboard operated by the opposite hand.

### Experiment 1

#### Familiarization

Prior to the main experiment, subjects took part in a set of familiarization trials (not illustrated) that used the same paradigm as in the subsequent acuity test (see next section). This block consisted of 50 trials which had larger frequency differences (2, 3, and 4 Hz) than those used later. Subjects received feedback on their performance following each trial. Familiarization continued until the subject scored eight correct trials out of the last 10; familiarization data were not collected.

#### Overview

The experiment involved a three-phase training session, which was preceded and followed by acuity tests (Figure 1B). The acuity test was designed to evaluate tactile discrimination capacity, whereas the training task was designed to induce feedback-guided learning that might affect acuity. Stimuli were applied to the right index finger. Subjects were told to treat training (Phases 2–4) and acuity testing (Phases 1 and 5) as independent experiments. To emphasize their independence, the instruction was given “The experiments will evaluate two different aspects of touch sensation.” The specific instruction for the training phase was: “Report whether the frequencies of the two stimuli feel the same or different.” As such it was a two-interval same/different task. The instruction for the acuity test was: “Select the stimulus with higher frequency. Even if the stimuli feel similar, indicate whether the first or second had higher frequency.” This was a two-interval, two-alternative, forced choice task. Since judgments made in a two-alternative, forced choice task are resistant to changes in decision criterion (see Macmillan and Creelman, 1991 for a review), any possible change in the subject's decision criterion during training should not directly affect performance in the acuity test.

#### Acuity test

The test required the subject to compare the frequencies of two vibrations, each of ~1 s duration with a 1 s inter-stimulus interval (Figure 1A). At the end of the second vibration, subjects indicated which vibration, first or second, they felt as having higher frequency. The purpose of the acuity test was to evaluate each subject's sensitivity in the neighborhood of 16 Hz, where sensitivity was defined as the Just Noticeable Difference (JND) above and below that frequency. At the start, the subject was presented with stimuli that differed by 3 Hz from the reference 16 Hz stimulus and were thus easily discriminable by all subjects. Using a staircase paradigm (Cornsweet, 1962), the comparison became more difficult by one step, with a 0.5 Hz step size, if the subject discriminated the pair of stimuli correctly three consecutive times; the comparison became easier by one step if the subject erred. In the case where both stimuli were 16 Hz, the subject's response was randomly assigned as correct or incorrect. The order of the two stimuli in a trial (16 Hz reference and comparison stimulus) was randomized. Separate measures of the JND were calculated for frequencies above and below 16 Hz; these are referred to as above-16 JND and below-16 JND. Trials of the above-16 and below-16 staircases were randomly interleaved. The acuity test consisted of 120 trials (60 trials for both the above-16 and the below-16 staircase) and the JNDs were calculated by averaging the last 12 peaks and valleys of the staircase diagram (Cornsweet, 1962).

The first acuity test is referred to as Phase 1, while the second test was at the conclusion of the experiment and is referred to as Phase 5 (Figure 1B). The training (Phases 2–4; see below) was carried out between the two acuity tests. The overall goal was to determine how training affected acuity.

#### Training paradigm

At the outset of training, each subject was randomly assigned to the Wide or Narrow condition. Trials in the training paradigm were structured differently from those in the acuity task. The subject was required to report whether the two consecutive stimulus trains had the same or different frequencies (two interval same/different task). The first stimulus was always a 16 Hz train of 1 s duration. It was followed by a 1 s inter-stimulus interval. The second stimulus was just under 2 s long (one period shorter than 2 s). The distribution of frequencies from which the second stimulus was taken was set up independently for each subject according to the JND obtained in the acuity test moments earlier. This ensured that the comparison stimuli were scaled in relation to that subject's baseline sensitivity. For this purpose, the individual's above-16 and below-16 JNDs were divided by two to define the size of the frequency steps. The full training stimulus set was composed of 17 stimuli with frequencies varying in one-step increments, thus covering a range, in Hz, from 16 – (4 × below-16 JND) Hz to 16 + (4 × above-16 JND). In each trial one of these stimuli was paired with the 16 Hz reference stimulus and the subject had to report whether the two stimulus frequencies were the “same” or “different.”

Training was composed of three successive blocks referred to as Phases 2, 3, and 4 (Figure 1B). Phase 2 consisted of 15 trials of each of the 17 different stimuli paired with 16 Hz (255 trials). In this phase there was no feedback and no points awarded; the results were used to evaluate the performance of each subject, across all frequency pairs. Phase 3 was the main training block. This phase consisted of 50 trials of each of the 17 different stimuli paired with 16 Hz (850 trials), but now with feedback. Subjects were instructed to perform the task in such a way as to maximize their final point count. The subject received points (displayed on the monitor) for each correct answer—answers that matched the computer's output on that trial—and lost points for each incorrect answer—answers that failed to match the computer's output on that trial. Unknown to the subject, the computer's feedback was biased rather than veridical: the probability of “same” feedback on each trial was drawn from a normally distributed function with a standard deviation (σ) twice as wide as the subject's JND (Wide condition) or else half as wide as the subject's JND (Narrow condition). We refer to the distance between 16 Hz and the second frequency presented on any given trial as the “single trial frequency difference” (STFD). The Wide and Narrow feedback distributions, in units of STFD, are illustrated in Figure 1C, together with the average performance across all subjects in the Phase 2 training block. The average response curve lies midway between the Wide and Narrow feedback distributions. In order to make sure all the subjects received an equal number of same/different feedback trials for each STFD, we drew the feedback for each trial from a pseudorandom pool without replacement, with same/different ratio fixed to comply to a normal distribution. This was done so to make sure we could confidently compare feedback effect across subjects.

Examples of the application of biased feedback are given in Table 1. In the Wide condition, a trial with STFD twice as large as the JND and thus reliably detected as different, was associated with “same” feedback with 0.60 likelihood and with “different” feedback with just 0.40 likelihood. In contrast, in the Narrow condition the equivalent trial was associated with “same” feedback with just 0.06 likelihood and with “different” feedback with 0.94 likelihood. On a trial with STFD equal to the JND, subjects in the Narrow condition received “same” feedback with 0.13 likelihood. On the same trial, a subject in the Wide condition received “same” feedback with 0.87 likelihood. The Narrow condition can therefore be considered as having approximately veridical feedback. It is important to note that the Wide and Narrow subject groups received the same set of sensory stimuli (normalized to their JND)—the only difference in training was the feedback following each trial during Phase 3.

Phase 4 was identical in procedure to Phase 2. There was no feedback and no reward points. The purpose of the block was to compare the subjects' response distribution with that in Phase 2. Any differences were attributed to Phase 3, the intervening training block that featured reward points and biased feedback.

#### Second acuity test

Phase 5 of the experiment was the second acuity test, performed in the same way as Phase 1, the first acuity test (Figure 1B).

#### Awarding of points

We anticipated that, during Phase 3, subjects might detect some imbalance in feedback and consequently show a tendency to give the easier answers—“same” in the Wide condition and “different” in the Narrow condition. To encourage subjects to attend to the stimulus pair on each trial, reward size varied according to the training condition. In Figure 1D the black and gray bars give the probability of the computer giving “same” and “different” feedback, respectively, as a function of the single trial frequency difference; the upper plot refers to the Wide condition and the lower plot to the Narrow condition. These probabilities were utilized for the weighting of rewards: the reward for the correct “same” responses was proportional to the sum of the areas of all gray bars, A_g_, while the reward for the correct “different” responses was proportional to the sum of the areas of all black bars, A_b_. In other words, the number of points awarded for a correct response was inversely related to the probability a priori of the computer providing that answer. By this formula, the product of the probability of a computer's feedback and the points gained by correctly matching that feedback were equal in the Wide and Narrow conditions.

The exact number of points awarded on each trial was defined as follows: for correct “same” response, the number of points equaled 17 times A_g_ divided by (A_g_ + A_b_). For correct “different” response the number of points equaled 17 times A_b_ divided by (A_g_ + A_b_). Filling in the values from the probability distribution function yields, for correct “same” responses, 7 points in the Wide condition and 14 points in the Narrow condition and for correct “different” responses 10 points for the Wide condition and 3 points in the Narrow condition. For both conditions, the penalty for incorrect responses was –9 points.

To understand the effect of the reward rule, consider the Wide condition. If the subject decided to respond “same” on most trials, many of those trials would be counted as correct, but would provide few points. In contrast, those trials in which the subject's response of “different” matched the feedback would provide more reward points. Since the subjects' goal was to amass points, a simple strategy of “always respond same” in the Wide condition would not be optimal, nor by the same token would a strategy of “always respond different” in the Narrow condition. An effective strategy for points accumulation would be to always report the stimuli based on the percept.

### Experiment 2

This experiment was designed to evaluate whether training had a unilateral or bilateral effect on acuity. To do so, two blocks were added to the acuity test. In these, the JND was evaluated before and after training not just for the right index finger as in Experiment 1, but also for the left index finger. The order of evaluating the JND on left versus right index finger was counterbalanced among different subjects.

### Data analysis

In order to quantify the magnitude of response distribution adaptation, a Gaussian function (Equation 1) was fit to each response distribution. To make the Gaussian fits comparable among subjects, the abscissa was drawn in units of step size of the individual's JND rather than stimulus frequency:

(1) $$P(same)=\alpha e^{-\frac{1}{2}{\left(\frac{\mu \;-\;d}{\sigma }\right)}^{2}}$$

where P(same) gives a probability density function that characterizes the subject's likelihood of judging the second stimulus as being the same as the first (16 Hz) stimulus, μ–d is the distance of the second stimulus from the peak of the distribution in units of steps (each step is one-half of JND; see above), and a, σ, μ are the parameters that give best fit to a Gaussian function. Specifically, a is the maximum probability of subject reporting “same,” σ is the standard deviation of the Gaussian function, and μ is the stimulus value associated with peak probability of “same” response.

To examine the overall effects (Figure 4), we computed for each subject the normalized JND change, as follows:

$$\text{normalizedJNDchange}=\frac{{\text{JND}}_{\text{Phase}5}-{\text{JND}}_{\text{Phase}1}}{{\text{JND}}_{\text{Phase}1}}$$

where the JND of each phase was the average of the JND values measured above and below 16 Hz.

In general we used nonparametric statistical tests which make no assumption about normality in data distribution. We used Wilcoxon signed-rank test and Wilcoxon rank-sum test to compare JNDs, the values of the Gaussian parameters and normalized JND changes. We used Pearson correlation to estimate correlation coefficients (Figure 4).

### Conflict of interest statement

The authors declare that the research was conducted in the absence of any commercial or financial relationships that could be construed as a potential conflict of interest.
